# Upper Extremity Thrombosis Presenting as Medial Elbow Pain after Shoulder Arthroscopy

**DOI:** 10.1155/2014/653146

**Published:** 2014-03-19

**Authors:** Moiz I. Manaqibwala, Irene E. Ghobrial, Alan S. Curtis

**Affiliations:** New England Baptist Hospital, 125 Parker Hill Avenue, Roxbury Crossing, MA 02120, USA

## Abstract

Deep vein thrombosis of the upper extremity is believed to be an uncommon complication of arthroscopic shoulder surgery. It most commonly presents with significant swelling and pain throughout the upper extremity. However the diagnosis can be easily missed when findings are more subtle and unrelated or the patient asymptomatic. In this study we report on 5 cases of postoperative upper extremity deep vein thrombosis (UEDVT). Each case was performed in the lateral decubitus position with an interscalene block and postoperative sling immobilization. All patients presented with a primary complaint of medial elbow pain and went on to require anticoagulation. Only one patient was found to have a heritable coagulopathy. The true incidence of thromboembolic phenomena after shoulder arthroscopy may be higher than that reported in the current literature. Therefore a high index of suspicion must be maintained when evaluating patients postoperatively to avoid misdiagnosis. Symptoms of medial elbow pain after immobilization in a sling should be considered an indication for duplex ultrasound evaluation. Ultimately, further prospective study is needed to better understand the prevalence, prevention, and management of this entity.

## 1. Introduction

Deep vein thrombosis (DVT) is a well-known complication following orthopaedic surgery. Most cases typically involve the lower extremities; however, 11% percent of all DVT are known to occur in the veins of the arm and forearm [1]. These upper extremity deep vein thromboses (UEDVT) are well described in the literature [2–5]. Almost 20% of primary cases are believed to occur sporadically and are considered idiopathic; the remainder occurs in the setting of Paget-Schroetter syndrome or venous thoracic outlet syndrome. The vast majority of cases, however, are secondary, caused by exogenous or endogenous risk factors including placement of central venous lines, malignancy, trauma, pregnancy, oral contraceptive use, ovarian hyperstimulation, and baseline coagulopathy [1, 2, 4, 5]. Shoulder arthroscopy was first described as a possible cause by Burkhart as early as 1990 [6]. At the time it was suggested there must be another underlying process other than the procedure, however, several subsequent case reports have refuted that claim [7–11].

Recently there has been increasing recognition that upper extremity injury and upper extremity surgery in general are associated with blood clot formation [12–15]. Dattani et al. in a systematic review showed a 0.52% rate of DVT in shoulder replacements, 0.64% in procedures for fractures of the proximal humerus, and 0.26% in replacement of the elbow [12]. The reported incidence of DVT following shoulder arthroscopy is <0.01%–0.38% as documented in several retrospective reviews [9, 11, 12, 16–18]. It is likely, however, that this is an underestimate of the true rate given that many cases may go undiagnosed as they are asymptomatic or present with nondistinct symptoms [2, 11]. Typically UEDVT presents as edema of the affected extremity (~80% of patients), pain (30–50%), and erythema (~15%) [1, 5]. Less common paresthesias, weakness, and visible venous collaterals are seen as well [4]. In this case series we seek to illustrate that even innocuous appearing medial elbow pain can be a primary presenting feature for UEDVT after shoulder arthroscopy.

## 2. Case Presentation

### 2.1. Case  1

A 58-year-old male (70 in, 185 lbs, BMI 26.5, nonsmoker) presented with right shoulder pain. After failure of conservative treatment for impingement, partial thickness rotator cuff tear, and acromioclavicular joint arthritis, he underwent arthroscopic subacromial decompression, distal clavicle excision, and debridement of the rotator cuff. At postoperative follow-up after 11 days, he presented complaining of pain and swelling at the medial aspect of his elbow. On exam, surgical sites were clean and dry; tenderness to palpation was present at his elbow area and he had pain with elbow range of motion. He was sent for duplex ultrasonography which showed partial occlusion with thrombus at level of mid to distal upper arm to antecubital fossa. He was treated with rivaroxaban (Xarelto; Bayer Schering Pharma, Berlin, Germany) 20 mg daily for 3 months. His elbow symptoms resolved rapidly and he was progressing with shoulder rehabilitation at his last follow-up 8 weeks after surgery.

### 2.2. Case  2

A 46-year-old male (76 in, 295 lbs, BMI 35.9, nonsmoker) with a known family history of coagulopathy presented with right shoulder impingement and partial thickness rotator cuff tear. Failing conservative management, he underwent subacromial decompression and debridement of the rotator cuff tear. On postoperative day 11, he reported pain in the inside of his elbow. On exam, surgical sites were healing well; however, at the medial elbow, a small area of swelling with exquisite tenderness to palpation was noted extending down the volar forearm. Duplex ultrasonography demonstrated thrombosis of the brachial vein and right basilic vein at mid-biceps to proximal forearm level. He was treated initially with enoxaparin (Lovenox; Sanofi-Aventis, Bridgewater, NJ, USA) and transitioned to warfarin (Coumadin; Bristol-Myers Squibb, Princeton, NJ, USA) for 3 months. At the last follow-up, 3 months after procedure, he had only mild shoulder pain with elbow symptoms resolved.

### 2.3. Case  3

A 43-year-old male (72 in, 200 lbs, BMI 27.1, nonsmoker) with a full thickness rotator cuff tear and impingement was treated with subacromial decompression and rotator cuff repair. On postoperative day 12, the patient presented with pain in the medial elbow. Surgical incisions were healing well; however, there was tenderness to palpation at his medial elbow with no other obvious findings. Patient was referred for duplex ultrasound, which demonstrated occlusive basilic vein thrombosis, as well as nonocclusive brachial vein thrombosis. He was treated with a course of aspirin 81 mg for 3 months. Elbow pain resolved within 3 weeks and he had excellent shoulder recovery at the latest follow-up 5 months after procedure.

### 2.4. Case  4

A 57-year-old diabetic male (68 in, 184 lbs, BMI 28, smoking ~1/2 pack daily) presented with 5 months of left shoulder pain from a full thickness rotator cuff tear. Eleven days after undergoing subacromial decompression and rotator cuff repair, he reported significant elbow pain. His examination demonstrated well healing portals without evidence of infection. However, there were slight swelling and localized tenderness over the medial elbow and proximal forearm. Duplex ultrasound revealed occlusive thrombus in the basilic vein with multiple nonocclusive thrombi in the brachial vein. Treatment included rivaroxaban 15–20 mg daily for a total of 3 months with resolution of symptoms within 3 days. At follow-up of 11 weeks, he was doing very well with no pain in his shoulder.

### 2.5. Case  5

A 66-year-old female (62 in, 149 lbs, BMI 26.8, nonsmoker) presented with 1 month of right shoulder pain. Her examination and imaging were suggestive of full thickness rotator cuff tear and tearing of the biceps. She underwent subacromial decompression, rotator cuff repair, and biceps tenodesis. Five days after surgery, she presented with severe pain in her operative arm mostly at the elbow with cold sensation in the fingers. On examination she had tenderness to palpation around the elbow with no other obvious findings. Nonocclusive thrombosis in the right brachial vein at the mid to lower arm was seen on duplex ultrasound. She was treated with rivaroxaban 20 mg for 3 months. At 1-month follow-up after surgery she had resolution of elbow symptoms and a normal postoperative shoulder examination.

All cases were performed arthroscopically in the lateral decubitus position with five to seven pounds of traction and general anesthesia after a preoperative interscalene block. There were no intraoperative complications. Patients who underwent rotator cuff repair were placed in a standard sling without pillow at all times, with instructions for daily active elbow, wrist, and hand exercises. Physical therapy was initiated at 7–10 days postoperatively. If no rotator cuff repair was performed, a similar sling was given, but for comfort only, with instructions to use the arm as tolerated. All DVT occurred on the operative side. Positive ultrasound resulted in a hematology consult in 4 cases and a vascular consult in one. Four hundred and ninety-six shoulder arthroscopies were performed during the 1-year period these cases presented.

## 3. Discussion

**Figure 1:**
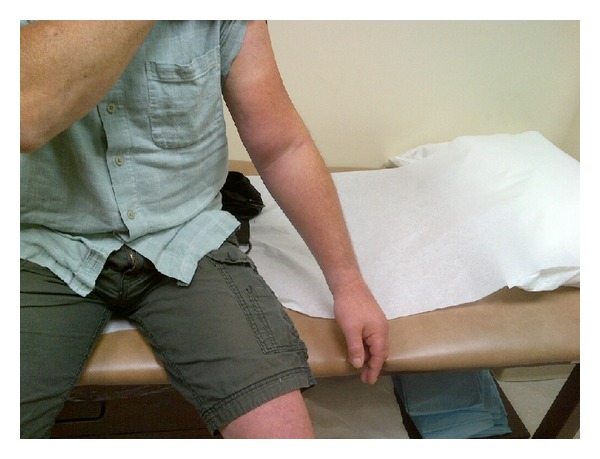
Typical presentation of upper extremity deep vein thrombosis with significant arm/forearm swelling and erythema.

Our case series illustrates how the presentation for UEDVT after shoulder arthroscopy can be fairly innocuous, and a greater incidence might be seen if a high index of suspicion is maintained. All of the patients in this report presented with medial elbow pain with variable degrees of localized swelling. The differential diagnosis for significant upper extremity swelling includes superficial phlebitis, cellulitis, contusion, muscle tear, intramuscular hemorrhage, gas gangrene, lymphedema, occult fracture, lymphangitis, allergy, and certainly DVT [8, 19]. Swelling is the most common presenting feature of DVT, whereas medial elbow pain is not a typical finding (Figure 1) [5]. Of the various cases in the literature, only two reports, by Bongiovanni et al., Delos and Rodeo showed patients presenting with this specific complaint [20, 21]. This may be because mild symptoms are sometimes dismissed as irritation from positioning or sling immobilization. However, as we maintained a low threshold for duplex ultrasound evaluation, we found evidence of DVT in five patients, four of whom were not found to have any other underlying cause or predilection.

Many factors related to the management of shoulder arthroscopy patients have been postulated to cause clotting; however, no good data exists. Bongiovanni et al. reported on three cases, which were diagnosed with heritable thrombophilia [20]. Only one of our patients, who had a family history of coagulopathy, was later diagnosed with a prothrombin gene mutation. Interscalene blocks and positioning in the lateral decubitus with the operative arm in traction have also been mentioned as a possible cause of thrombosis. This was the case with all of our patients as well as 6 cases reported by Kuremsky et al. [11]. A systematic review by Dattani et al. also alluded to positioning as a cause. They found that in 18 cases of DVT after shoulder arthroscopy 14 patients were in the lateral decubitus position and 4 sat beach chair [12]. Although far from conclusive, this data may suggest a higher predisposition when being lateral. Several other theories exist; Polzhofer et al., in one case, speculated on compression of the subclavian vein by the arthroscopic shaver as the likely cause versus compression from inadequate positioning of the arm or fluid-induced subcutaneous swelling [22]. Garofalo et al. suggested the need to study intraoperative traction weight, procedure duration, and postoperative immobilization as possible causes [9]. Plaster cast immobilization of the arm has been shown in one study to increase the rate of UEDVT [23]. Perhaps, this can be extrapolated to sling immobilization as well. In our series all patients received slings, and although some were for comfort only, they were typically used in the initial period as a result of immobility from the interscalene block. Given most of our cases involving the basilic vein and all presentations involving the medial elbow, we would suggest that perhaps a pressure phenomenon between elbow and chest wall or pillow in the sling may contribute to venous occlusion and thrombosis as well.

Although the absolute cause in many cases remains unknown, diagnosing UEDVT remains important. The reported rates of diagnosed DVT after shoulder arthroscopy are between <0.01% and 0.38%. However, it is widely acknowledged that these low rates are probably underestimates [9, 11, 12, 16–18]. All studies to date have been case reports or retrospective reviews and therefore are subject to reporting issues and information bias. These studies were also likely to miss mild or subclinical cases, which can represent a large percentage as seen in the literature on indwelling catheters where an approximately 60% rate of DVT has been shown to be present on routine screening [2–4, 11]. In addition, evidence related to total shoulder arthroplasty (TSA) also suggests that the true incidence of UEDVT is likely higher. Initially, believed to have a venous thromboembolism rate of 0.01%–0.52%, a recent study by Willis et al. demonstrated that, upon prospective evaluation of TSA, the rate of DVT was actually closer to 13%, with a 3% rate of pulmonary embolus (PE) [14, 17]. The clinical relevance of this finding is significant as the manifold higher than the reported rate of DVT led to a high rate of PE. This was consistent with previous work showing that up to 36% of UEDVT goes on to PE and it ultimately raises the question of chemical prophylaxis [2]. No studies related to shoulder arthroscopy have evaluated prophylaxis and no guidelines exist. However, when Jameson et al. treated all inpatients after TSA with enoxaparin, following UK National Institute for Health and Clinical Excellence guidelines, although numbers were small, they found a significant decrease in the rate of PE [17]. There are obvious differences between TSA and shoulder arthroscopy, and it would be difficult to advocate this type of protocol based on current evidence, but it is important to recognize the risks associated with shoulder arthroscopy and the need for further study.

In summary, UEDVT after shoulder arthroscopy, although an uncommon phenomenon, is probably more prevalent than currently reported. Diagnosis requires a high index of suspicion and when symptoms unrelated to the shoulder are present, especially medial elbow pain, duplex ultrasound evaluation should be considered. Ultimately, further study is needed to prospectively evaluate the true incidence of UEDVT and to determine what if any prophylaxis is indicated.
